# A High-Energy-Density Magnesium-Air Battery with Nanostructured Polymeric Electrodes

**DOI:** 10.3390/polym14153187

**Published:** 2022-08-04

**Authors:** Abdulrahman Faraj Alharbi, Abdulaziz Abdulkarim Mansour Abahussain, Mian Hammad Nazir, Syed Zohaib Javaid Zaidi

**Affiliations:** 1 Department of Chemistry, Collage of Science and Humanities, Shaqra University, Al Quwayiyah 19257, Saudi Arabia; 2 Department of Chemical Engineering, King Saud University, Riyadh 11451, Saudi Arabia; 3 Faculty of Computing Engineering and Sciences, University of South Wales, Treforest, Pontypridd CF37 1DL, UK; 4 Laboratory for Energy, Water and Healthcare Technologies, University of Punjab, Lahore 54590, Pakistan; 5 Institute of Chemical Engineering and Technology, University of Punjab, Lahore 54590, Pakistan

**Keywords:** Mg-air battery, air electrode, nanostructured polymeric electrodes, capacitance, charging, discharging, energy density

## Abstract

The greenhouse emissions are biggest challenge of the present era. The renewable power sources are required to have characteristics of good charge capacity, energy density with proven charging discharging cycles for energy storage and applications. Mg-air batteries (MABs) are an alternative renewable power source due to their inexpensive cost. In particular, the previous reports presented the metal-air battery structure, with a specific energy overall output of 765 W h kg^−1^. This paper is focused mainly on the MAB, which employed nanocomposite polymeric electrodes with a proven energy density of 545 W h kg^−1^ and a charge capacity of 817 mA h g^−1^ when electrolyzed at a cycling current density of 7 mA cm^−2^.

## 1. Introduction

Metal-air batteries exhibit greater energy density and have improved efficiency in different energy storage application. These batteries require improved cell design with the use of active metals to fulfil the capacitance for the energy requirements of stationary and moving appliances. These batteries were found to be inexpensive and hydrophobic with lower sensitivity to moisture in comparison with lithium and sodium [1,2]. These batteries have an ability to recycle the aqueous media when needed. The traditional lithium in aqueous media causes safety hazards with greater chance of provision. The use of expensive surfactant and porous layers makes these batteries unaffordable [3]. Metal-air batteries such as zinc and magnesium-air batteries have no safety issues and have been found to be attractive candidates for rechargeable batteries. The theoretical electrode potential of 1.65 volts was reported for zinc batteries [4], which is higher than the electrode potential of magnesium-air batteries of about 1.3 volts.

Metal-air batteries may form complex dendrite structures due to the flow of electrolytes over the electrode surface due to the large cycling time and repeated secondary reactions on surface of the anodic electrodes [5]. This issue can be prevented by using a catalyst, which could increase the lifetime discharging cycles and limit the secondary reactions at the electrode surfaces. This may increase the mass transport between the electrolyte and electrode surface, which ultimately improves the charge transfer, limiting formation of dendrites over the electrode surfaces [6]. All secondary reactions involving OER and ORR remain in the oxide state rather than being immersed in the electrolyte, as in the metal ion–air batteries [7,8]. This leads to the formation of undivided batteries with null requirement of the membrane structure to get rid of metal particles migrating toward the anode and ceases the movement of electrolyte circulation within the battery in an orderly manner, to have a compact electrodeposition of metal ions during the charging phase rather than removing the heat and maintaining the level of the electrolytes [9]. 

Metal-air batteries filled with electrolytes have a strong alkaline aqueous solution with a conductive nature to accelerate redox species over a catalytic surface of both electrodes [10]. The dissolution capacity of metal oxide species in alkaline solution is minimum as compared to strong acidic media, which facilitate the durability of both electrodes in the closed environment. It is pertinent to mention here that the catalyst present on the surface of an air electrode is immersed in the electrolytic solution; however, the air diffusion surface maintains a nil electrolyte presence to provide smooth air circulation over the catalytic surface, thereby having reasonable mass transport [11].

The metal oxide electrode undergoes two-step oxidation over the discharging phase with two electrons transferred in the metal-air battery [12,13,14,15]:

M + 2OH^−^ ⇆ M(OH)_x_ + 2e^−^ (1)

M(OH)_x_ + XOH^−^ ⇆ MOx + XH_2_O + 2e^−^ (2)

The overall cell reaction is then:

XM + 2O_2_ ⇆ MOx (3)

where M denotes the metal ions.

Recent developments about the metal-air batteries have reported overall energy densities of above 100 W h kg^−1^, which seems reasonable compared with other energy storage devices such as metal acid batteries, which reported energy densities above 45 W h kg^−1^ [15,16]. This is very obvious that the litertaure has always reflects a gap for achievements and improvements to increase the energy density and capacitance values for metal-air batteries. Metal-air battery performance is dependent upon anode, and reactions take place at the anodic side. The use of a bi-functional catalyst in air electrodes is one of the drawbacks of the occurrence of side reactions at anodic surfaces at lower overpotential values [17]. The requirement of the anode should be likely to have the capacity to bear corrosion during oxygen evolution reaction and start secondary reactions at a very high positive voltage [18]. The slow rate of both oxygen evolution and reduction reactions mainly decreases the energy density [19,20,21,22]. This may cause rapid erosion and damage of anodic and cathodic electrodes during the redox reaction and electron transfer process in metal-air batteries [23,24,25]. In certain circumstances metal-air batteries are better, with reported theoretical energy densities greater than 3500 W h kg^−1^ with repeated cycling of 18 batches [26]. With a proven family of good efficiency and capacity of metal-air batteries, the MAB is potentially reported to be a better alternative in comparison to LIBs, with an overall volumetric capacity of more than 3800 mAh·cm^−3^, which is about five times greater than that of LIBs. Practically, the use of the organic electrolyte leads to the energy density of 746 W h kg^−1^ with average cycling potential of 2.4 V vs. Mg^2+^/Mg [27]. The use of nanoparticle-based palladium over metal-based organic substrate has proved to be high-strength with reported charging/discharging cycles of more than 900 by applying a current density of more than 10 mA cm^−2^. The hydrogen evolution reaction was reported at 0.1 V vs. Hg/HgO for this cathodic material in alkaline solution, which seems to be a good support for metal-air batteries for cathodic compartment [28].

This paper provides a report related to the energy density of a Mg-air battery (MAB), having an advanced type of assembly of cell structure and novel polymeric materials that has demonstrated an energy of 545 W h kg^−1^ with proven a highest capacitance of 817 mA h g^−1^ when electrolyzed for a full cycle of 17 h at an applied current density of 7 mA cm^−2^.

## 2. Experimental Details

### 2.1. Manufacturing of Electrode for Cathodic Compartment

The Pd catalyst was prepared by a colloidal suspension reduction procedure, using chloride as a complexation agent [29]. The carbon polymer support was held suspended via clamps in 70% nitric acid at 110 °C for 1 h. Subsequently after this acidic treatment, the carbon polymer support was thoroughly cleaned/washed and left in distilled water over the night and further dried at 90 °C in a hector oven. Furthermore, the obtained substrate was subjected to a clean jaw crusher to convert it into coarse powder, and by sieving unit operation an appropriate amount of powdered carbon polymeric substrate was conceived and separated in a collection petri dish. The obtained powder was immediately utilized for colloidal suspension reduction. Here an acidic media containing a known amount of PdCl_2_ was put on to the polymeric powder semi solid to obtain a catalytic mass loading of 20 wt% of Pd over the polymeric powder substructure. The Pd chloride solution decomposes by addition of an appropriate quantity of peroxide, and then the acidic nature was maintained by adding 70% nitric acid (Fisher scientific) drop wise at a pH of 4.7 to obtain a PdOx/C polymeric slurry. The obtained oxide of metal was readily converted via H_2_ stream at an ambient temperature (25 °C) to obtain a 20 wt% of reduced Pd/C component.

### 2.2. Manufacturing of the Electrode for Anodic Compartment

The Magnesium oxide–based anodic electrode was prepared by a dried heat treatment procedure [30] moreover the Mg forerunner MgCl_2_ was immersed in an alcohol via constant stirring at 700 rev. min^−1^ with the immediate addition of the necessary amount of NaNO_3_ and 0.2 mol dm^−3^ TBAOH. The mixture was kept under optimum conditions in a closed environment to dissolve and set by evaporation overnight, and the obtained slurry was heat treated at 550 °C for 60 min, the resultant MgOx being exposed to the nitrogen environment for any leftover species during heat treatment, and then taken to the grinding–milling unit operation in alcohol for 36 h as described in [31]. The MgOx was crushed with the leftover carbon polymeric powder in the grinding–milling unit operation for 6 h with the dilution of alcohol to comply with the maximum transformation of added chemicals in the MgOx powder. The MgOx catalyst exhibited a uniform coating to apply over the current collector.

### 2.3. Assembling of Cathodic Electrodes

The manufacturing of the cathodic compartment of the gas diffusion anode contains three important pillars joined closer by a heat treatment pressing machine with a air absorption layer, a Pd/C component as a catalyst surface and a current collecting element. The gas absorption layer was developed by mixing 70 wt% of well defined surface area of 47 m^2^ g^−1^ with graphite submerged with 20 wt%. PTFE solution by RS UK and 1 cm^3^ alcohol per 0.5 g of powder reagents until a thick slurry was obtained. The slurry was spread and coated uniformly over a 4 cm × 4 cm piece of carbon polymer with the thickness of 0.1 mm, treated with 70% nitric acid (Fisher scientific) at 110 °C for 1 h, mixed constantly and finally hot pressed at 190 °C and 150 kPa at a time interval of 7 min to a layer of approximately 0.5 mm.

The Pd/C compnent as catalytic layer was added with 5 wt%. Nafion (Sigma Aldrich, St. Louis, MO, USA) in an alcohol solution that was stirred for 2 h. The stirring formed a thick, viscous, black slurry that was uniformly coated over the gas absorption surface. Subsequently, a piece of solid nickel sieve mesh 4 cm × 4 cm was cut, and the catalyst layer was coated over the top sieve. The air electrodes for cathodic compartment with three layers were heat treated pressing for 5 min at 240 kPa and 170 °C.

The component was wrapped using a baking wrap to get rid of erosion of the electrode due to the extreme temperature of the pressing machine. The electrode was simultaneously removed and dried with nitrogen gas to remove any dirt and left overnight at optimum conditions.

### 2.4. Assembling of Anodic Electrodes

The anodic MgOx electrodes based on 90 wt.% active MgOx prepared as described in Section 2.2 with 5 wt.% carbon powder strongly stirred together with 5 wt. % PTFE solution provided by DISP 40 solution to form a thick slurry were spread and coated over a 4 cm × 4 cm piece of carbon polymeric structure with the thickness of 0.1 mm, treated with 70% nitric acid (Fisher Scientific, Hampton, NH, USA) at 110 °C for 1 h and mixed constantly over a water bath at 70 °C and dried over night. Further, the thick slurry containing MgOx was hot pressed between two steel mesh current collectors cut at length of 4 × 4 mm^2^ at 170 °C and 70 kPa for 7 min to a layer the thickness of 0.5 mm.

### 2.5. MAB Battery Electrochemical Testing in an Advanced Configuration

A Mg-air battery (MAB) having a 4 cm × 4 cm gas diffusion electrode as synthesized in Section 2.4 was held in front of the MgOx electrode while immersed with 70 cm^3^ of 7 mol dm^−3^ NaOH as an electrolyte in the cathodic compartment. The electrochemical measurements such as charging, discharging, chronoamperometry and other studies were conducted using Ivium-based Potentiostat provided by Alvatech UK. Both compartments were connected with the connections of Potentistat. The Hg/HgO reference electrode was immersed in between the cathode and anode PTFE tube via 20 cm, having a diameter of 1 mm. Air was supplied through an air pump consitently into the drift patterns of the cell at a flowrate of 0.5 dm^3^ min^−1^ via an air pump with twin inlets.

The electrochemical characterization was performed by charging the MAB at a constant current density of 10 mA cm^−2^ to a capacitance of 1100 mA h g^−1^ and was cycled for discharging at varying current densities ranging from 7–25 mA cm^−2^ to measure the outcome of current density over the cell charging capacitance accompanying energy densities. The electrodes were tested to varying capacitance in the range 750–1025 mA h g^−1^ and further discharged at 525–680 mA h g^−1^ to obtain the incremental change in the discharge capacity. The constant current electrolysis was performed to measure the tendency of oxygen evolution and reduction reactions at of 23 mA cm^−2^ of an applied current density. The electrodes were then removed from the system, and the battery was cycled at 10 mA cm^−2^ to a capacitance of 1100 mA h g^−1^ and cycled for discharging at a 20–1000 mA cm^−2^ current density for 17 cycles.

### 2.6. Blueprint and Assembling of MAB

**Figure 1:**
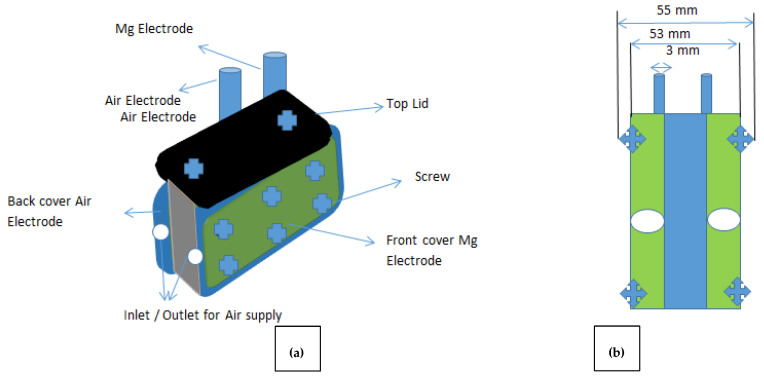
MAB configuration and arrangements: (**a**) overall schematics of the battery reflecting the electrode connections with air entry and exit points; (**b**) frontal view showing the internal dimensions of the geometry of the battery.

The dimensions of the MAB was 55 mm × 53 mm × 3 mm with an equivalent mass of 175 g and the tendency to handle up to 70 cm^3^ of electrolytic solution. The Mg and air electrodes, both 17 g, were maintained at a distance of 3 mm. The anode was inserted into one side of the MAB while the air electrodes were placed at the other edge; however, the main assembly was protected by two 5 mm polymeric type gaskets obtained from RS UK. The electrode connections were maintained in a square type of assembly to maintain minimum short circuit chances. The PTFE-based tube was inserted in the flowfields to supply oxygen or air. The battery was sealed using polymeric fibre based screws to ensure zero leaks of the MAB. The overall system of the closed loop battery is shown in Figure 1.

## 3. Results and Discussion

### 3.1. Anodic Electrode Characterization

**Figure 2:**
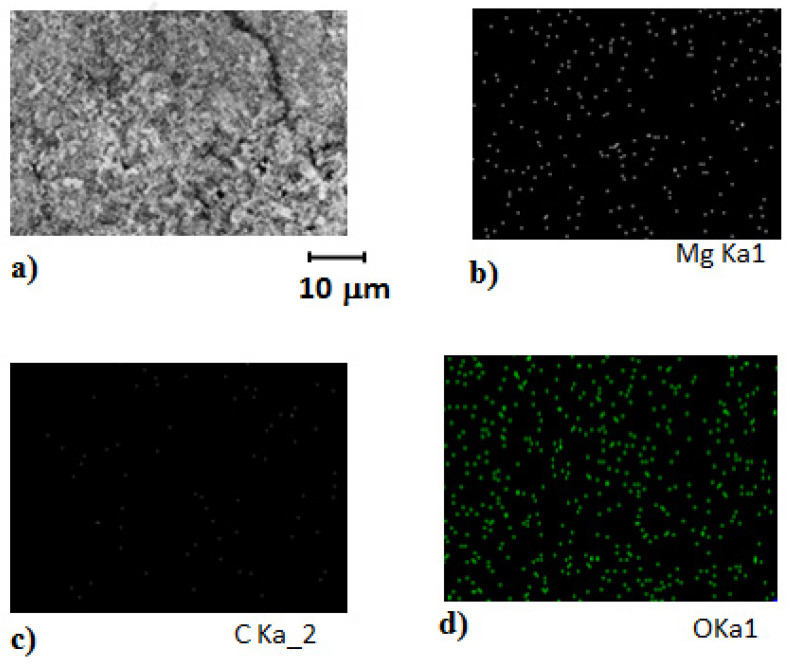
Internal morphology and structural properties of a MgOx electrode over a conductive polymeric base by (**a**) SEM, (**b**) EDX for Magnesium, (**c**) for Carbon, (**d**) for Oxygen after hot pressing.

The MgOx electrode was characterized by using scanning electron microscopy. The results in Figure 2a represent a well-structured formation of Mg over the surface of the closed pattern with the presence of uniform particles over the polymeric structure as a substrate material. Further EDX results shown in Figure 2b–d demonstrate the presence of Mg in a reflective manner covered over the polymeric carbon surface. 

### 3.2. Performance Characterisation of MAB

**Figure 3:**
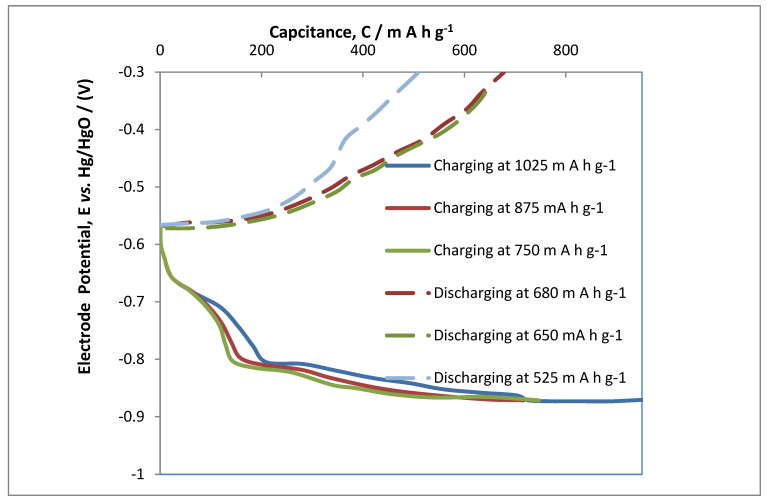
Cycling of MAB at capacities of 750, 875 and 1025 mA h g^−1^ with discharging at 680, 650 and 525 mA h g^−1^ at ambient conditions in alkaline media having 7 mol dm^−3^ NaOH as an electrolyte.

Figure 3 represents the charging and discharging capability of the magnesium electrode in the MAB at 25 mA cm^−2^ of current density. The cell was charged at different capacities during the enhanced cycle of about 17 at a 750, 875 and 1025 mA h g^−1^. The charging curves showed that the optimum charging capacities were found to be at capacity of 875 mA h g^−1^, because providing further charge of 1025 mA h g^−1^ showed no flauctution and any further rise in charging functioning and performance; rather, a negligible change in voltage charging efficiency was observed. Different capacities were applied in the ranges of 680, 650 and 525 mA h g^−1^. The discharge capacity was found to have a slight plateau in voltage closer to 525 mA h g^−1^. This phenomenon is usually due to oxygen evolution and secondary reactions, which also effects the overall performance of Columbic efficiency in the MAB. The performance of the Mg-based electrode is found to be diminishing, especially based on the hypothesis that the experimental outline here were carried out at increasing current densities than reported in other peer reviewed research work. As an example, in previous studies reduced graphene–based anodic electrodes had an improved capacity for the active composite at a much lower current density of 10 mA cm^−2^ in comparison to the much higher applied current densities 25 mA cm^−2^ in this study, which provide the activity and strength of MgOx electrodes utilized for MAB at these higher current densities [32].

### 3.3. Cathodic Performance of Air Electrodes in MAB

**Figure 4:**
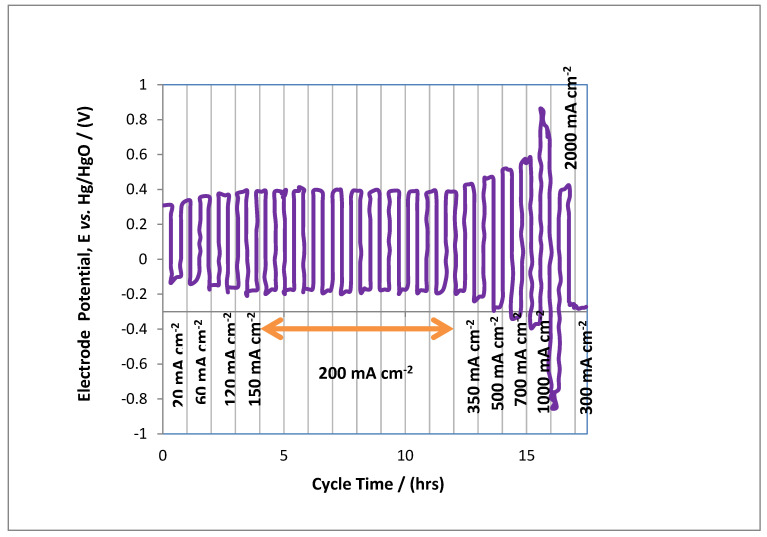
Cycling performance of a MAB at 20, 60, 120, 150, 200, 350, 500, 700, 1000 and 2000 mA cm^−2^ of current densities. The cycling time was intervals of 60 min.

We obtained by hot press a cathodic air electrode with a geometrical area of about 16 cm^2^ with the defined thickness of 0.7 mm. Further details of air electrodes are given in Section 2.3. The charging discharging cycles are represented in Figure 4, which were reported at different values between 20 to 2000 mA cm^−2^; this represents the strength and capability of synthesized gas diffusion electrodes at greater current density. The cycles were increased for about 17 h at a current density of 350 mA cm^−2^, and it was found that an air electrode is strong enough to withhold high oxidizing current densities; in contrast, at >400 mA cm^−2^ air electrodes were starting to be affected by strong oxidization reactions with secondary reactions due to strong overpotential at the anodic side of the MAB, which ultimately ruptured or destroyed the electrode structure. This is all due to excessive oxygen evolution. Further increasing cycling at current density of 2000 mA cm^−2^ showed that the gas diffusion electrode was actually desecrate. This trend indicates the decrease in potential to −700 mV vs. Hg/HgO, which conforms the deterioration of catalysts of due to oxidation of the current collecting material. This phenomenon reveals that the optimum operative conditions for a MAB were found to be at 350 mA cm^−2^ current density, as this applied condition can withstand rapid charging/discharging and would not affect overcharging of the MAB. The carbon support provides good catalytic loading for oxygen evolution and side reactions; this provides a sustainable compromise of palladium for a secondary reaction [28].

### 3.4. Performance Capacity Charge/Discharge of MAB

**Figure 5:**
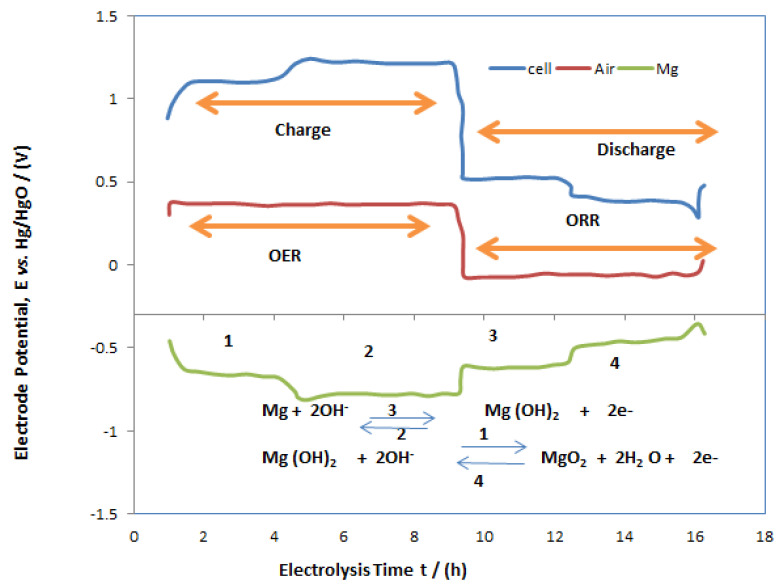
Cycling for discharge profile of the MAB after cycling at 17 mA cm^−2^ for 17 h at 7, 13, 17, 23 and 25 mA cm^−2^ current densities with an air inlet flow of 0.5 dm^3^ min^−1^.

**Figure 6:**
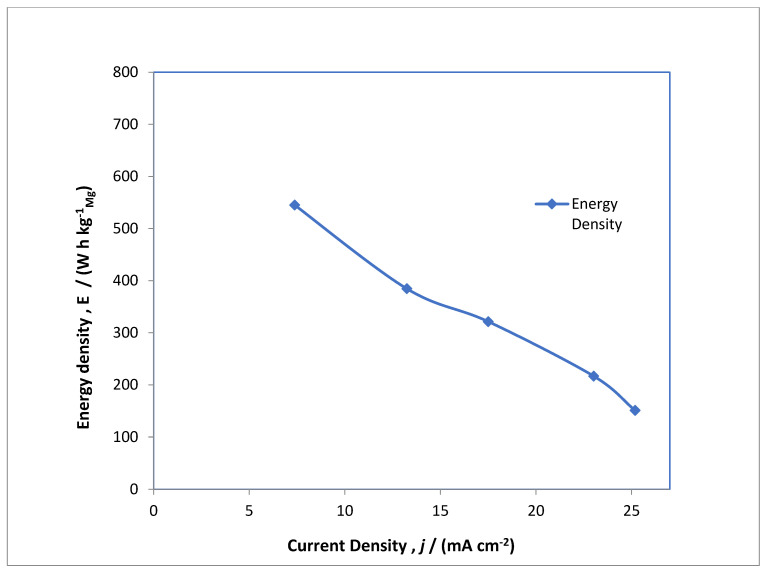
Energy density profile for a MAB at applied current densities of 7, 13, 17, 23 and 25 mA cm^−2^.

**Figure 7:**
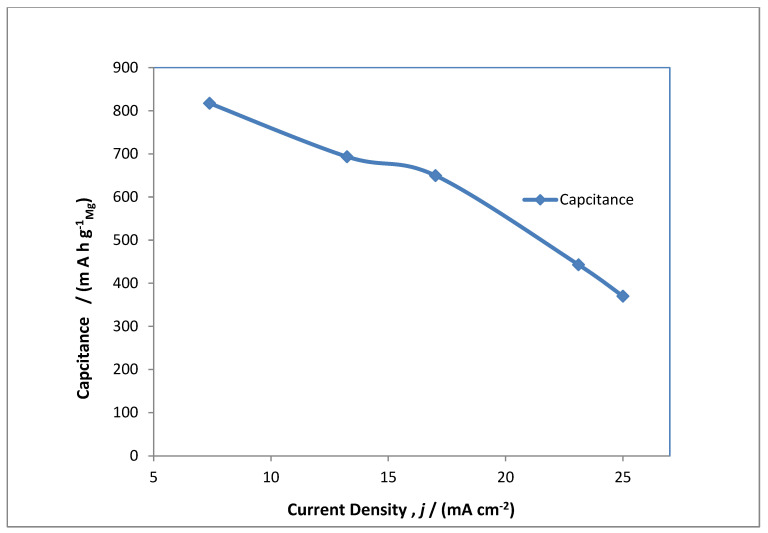
Charge capacitance profile for a MAB at applied current densities of 7, 13, 17, 23 and 25 mA cm^−2^.

A typical electrolysis profile was conducted for the MAB at 20 mA cm^−2^, which is shown in Figure 5 as charge/discharge studies. The performance of the MAB oxygen evolution and subsidiary secondary reactions are observed at the anodic side of battery at 0.35 V vs. Hg/HgO whereas MgOx transforms via oxygen reduction reaction at approximately −0.5 V vs. Hg/HgO to hydroxide of magnesium as an intermediate product. During discharge, which takes place at the air electrode, depicted with the red line, reduction takes place at −0.3 V vs. Hg/HgO, and MgOx is converted into Mg via hydroxide of magnesium at −0.7 and −0.5 V vs. Hg/HgO. This phenomenon showed double redox reaction at the magnesium electrode, which showed two distinctive plateaus in the MAB charge/discharge studies. From this observation it can be understood that overwhelming the hydrogen evolution over the MgOx electrode deteriorated the rate efficiency of the MAB. This seems very anticipated as the conversion of magnesium hydroxide to magnesium and the hydrogen evolution reaction are competing. In order to have inhibition of the hydrogen evolution reaction, different peers have explained that the application of other sulphur-based catalysts in the electrolyte and in the prepared MgOx electrolytic surface had favorable outcomes over competing reactions [33]. Futhermore, an intrinsic distinctive feature of the MAB is the extremely large potential drop between charging and discharging of cell. As shown in Figure 5, the blue line, the large potential drop is mainly due to anodic reactions at the air electrode, and the reason behind it is the occurrence of a minimum rate of reaction for the oxygen evolution reaction and some secondary reactions 0.3 V vs. Hg/HgO. This drawback cannot be controlled easily and is also reported by previous authors that most gas diffusion electrodes in metal-based batteries have a much greater voltage fluctutaions between charging and discharging cycles [34]. Figure 5 depicts the charging of the MAB by cycling at different current densities in the range of 7 to 25 mA cm^−2^. The electrolysis was conducted for about 17 h for each cycle of applied current densities, and the discharge profile summarizes from each cycle for different current densities. There is a sharp decrease in electrode potential after 11 h of electrolysis at a current density of 7 mA cm^−2^, and after completion of electrolysis the potential is decreased to 0.2 V vs. Hg/HgO. This phenomenon arises due to slow kinetics of oxygen evolution reaction at the anodic side of MAB. The similar phenomenon is observed while increasing the current density to 25 mA cm^−2^. This action is also reported by many authors in metal-air batteries [35]. Figure 6 shows the energy density, and Figure 7 shows the capacitance obtained from cycles during the constant current electrolysis at the current densities ranging from 7 to 25 mA cm^−2^. There is an ambiguous trend fashion in both characteristics with the applied current density as the energy density reduced from 545 to 151 W h kg^−1^ on enhancing the current density from 7 to 25 mA cm^−2^, and similar trends were observed for capacitance from 817 to 370 mA h g^−1^. The energy density and capacitance obeyed an inverse non-linear relationship with applied current densities until 25 mA cm^−2^, which is due to competing secondary reactions on both electrodes. 

**Figure 8:**
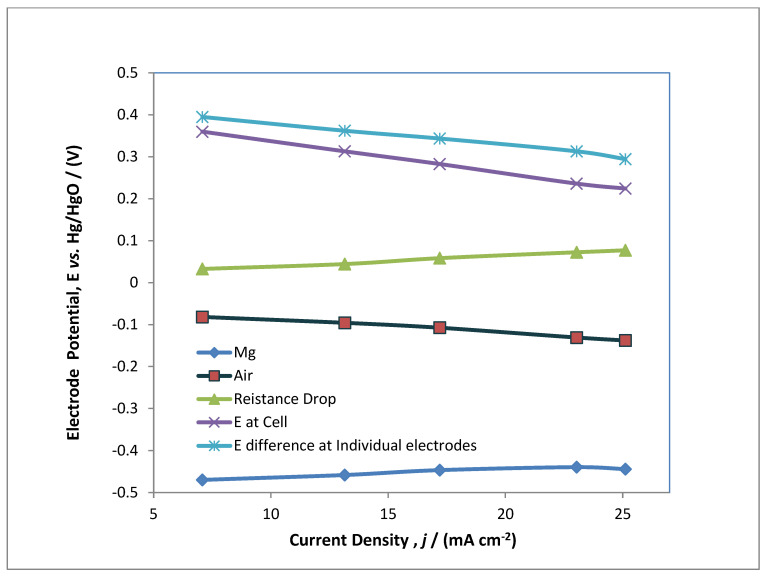
The variation in individual electrode potentials for Mg, air, resistance IR drop and cell potential at increasing applied current densities.

Figure 8 reports the variation among electrode potential for *E*_Mg_ (blue) and *E*_Air_ (red) vs. the Hg/HgO reference electrode, *E*_cell_ (purple), which is the measured cell potential, while *E* (sky-blue) denotes the difference *E*_Air_ − *E*_Mg_, whereas *E* resistance drop denotes (green) IR drop due to ohmic resistance. The result denotes that electrode potential (denoted by blue) is slightly decreasing due to side reactions of hydrogen evolution at the cathodic side, whereas for the air electrode the electrode potential remains almost the same due to ohmic drop. The ohmic drop obeys Ohms law and provides resistance due to presence of electrolytes as reported in different articles [21]. The variation in electrode potential between individual electrode is found to be decreasing. This is due to geometrical area of electrodes and the inter-electrode gap between the anodic and cathodic electrodes. However, the electrode potential at the cell is decreasing due to the resistivity of the aqueous solution present in the battery. These values of electrode potential are compared with varying current densities ranging from 7 to 25 mA cm^−2^. The overall results showed in Figure 8 denotes that current density has a nearly inverse power relationship with electrode potentials. This might be due to the minimum order of reaction for the oxygen reduction reaction and oxygen evolution reaction over the time of the electrolysis.

### 3.5. Electrode Characterisation after Cycling of MAB

**Figure 9:**
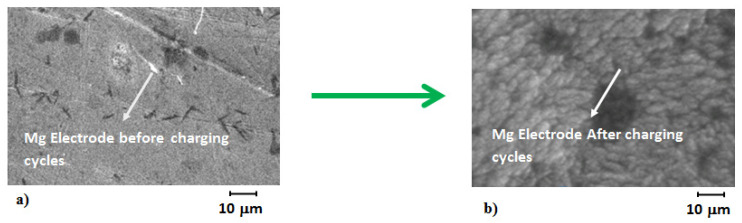
MgOx electrode micro-graphs by SEM (**a**) before and (**b**) after 17 cycles.

**Figure 10:**
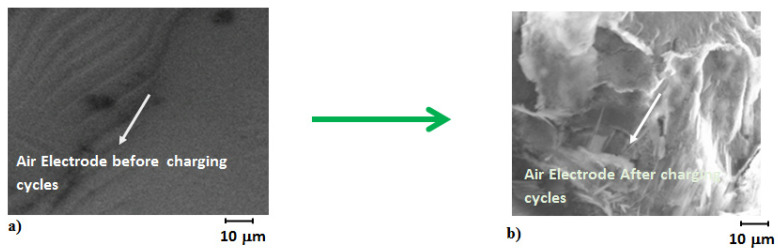
Air electrode micro-graphs by SEM (**a**) before and (**b**) after 17 cycles.

In order to report the strength exhibited by magnesium and air electrodes after electrolysis of about 85 h, SEM micro-graphs were conducted to observe the morphology of electrodes before and after the electrolysis cycle (Figure 9). Before electrolysis the as-obtained MgOx electrodes were uniform with defined structures, and after cycling during electrolysis the electrodes become full of particles from around 20 to 70 nm to 200 nm with fuzzy spherical shape. This might be due to the fact that particle size increases due to dissolution and raping of MgOx particles. Due to this, a greater mass of MgOx catalysts becomes inactive for an electrochemical side reaction, which results in the gradual deterioration of the magnesium electrode. This is likely to be a postulate for the decrease in capacity and energy density during 85 h of electrolysis. It was observed that the paste containing the magnesium electrode was poured out from mash, and also, very minuet bubbles of hydrogen were seen over the magnesium electrode morphology, which causes removal of active mass from the magnesium electrode. This might be another reason in the loss of energy density and capacitance of the MAB. In case of the air cathode (Figure 10) significant particles size were observed over the surface with prismatic types of structures, which showed that palladium carbon particles were precipitated due to oxygen evolution reaction and side reactions that take place during anodic compartment activity. The surface structure was found with slight raping and petting, which might be due to catalysts particles, and it deteriorated during charging and discharging cycles.

## 4. Conclusions

The main aim of this research work was to measure the electrochemical characterization of the MAB. The energy density of about 321 W h kg^−1^ at a current density of 17 mA cm^−2^ was observed considering the total mass of active electrodes without electrolyte and electrical factors such as inbuilt connections. The MAB was observed to be strong enough to control greater current densities up to 350 mA cm^−2^ and operated between a stable discharge potential of 0.4–0.1 V vs. Hg/HgO. The MAB also reported the capacitance of 649 mA h g^−1^ at 17 mA cm^−2^, which is quite comparable with the other reported values in the literature. Although there is a problem of oxygen evolution reaction and hydrogen gas bubbles, which undergo materials deterioration during the electrolysis cycle at the anodic and cathodic compartments, future catalysts design for the MAB should involve inhibition of hydrogen evolution and a rise of the potential at which oxygen evolution takes place. This can be done by improving electrode design by adding sulphide components. Greater mass transport can be achieved by using 3D electrodes with improved porosity and defined structures of flowrates.
